# RKIP overexpression reduces lung adenocarcinoma aggressiveness and sensitizes cells to EGFR‐targeted therapies

**DOI:** 10.1002/1878-0261.70096

**Published:** 2025-07-28

**Authors:** Ana Raquel‐Cunha, Joana Pinheiro, Rui F. Marques, Patrícia Fontão, Diana Cardoso‐Carneiro, Adriana Mendes, Izabela N. F. Gomes, Ana Carolina Laus, Renato J. da Silva‐Oliveira, Rui Manuel Reis, Olga Martinho

**Affiliations:** ^1^ Life and Health Sciences Research Institute (ICVS), School of Medicine University of Minho Braga Portugal; ^2^ ICVS/3Bs‐PT Government Associate Laboratory Braga/Guimarães Portugal; ^3^ Molecular Oncology Research Center Barretos Cancer Hospital Brazil

**Keywords:** EGFR‐targeted therapy, lung adenocarcinoma, non‐small‐cell lung cancer, Raf kinase inhibitory protein, therapeutic resistance

## Abstract

Lung adenocarcinoma (LUAD), the most common subtype of non‐small‐cell lung cancer (NSCLC), is often driven by mutations, particularly in epidermal growth factor receptor (*EGFR*), that guide targeted therapy choices. However, resistance to these treatments remains a major clinical challenge. Raf kinase inhibitory protein (RKIP), encoded by the *PEBP1* gene, a metastasis suppressor, modulates key oncogenic pathways and may influence tumor aggressiveness and therapy response. Yet, its specific role in NSCLC remains unclear. This study investigates the influence of RKIP expression on NSCLC aggressiveness and explores its impact on therapy response, particularly to EGFR‐targeted therapies. *In silico* analyses revealed that lower *RKIP* mRNA expression correlates with poorer survival outcomes in LUAD patients but not in other NSCLC subtypes. Genetic modulation of *RKIP* expression in LUAD cell lines demonstrated that its overexpression reduced migration, spheroid integrity, and suppressed tumor growth, whereas RKIP knockout had opposite effects, particularly *in vivo*. Expression profiling showed that RKIP overexpression impacts the activation of mitogen‐activated protein kinase (MAPK), RAC serine/threonine‐protein kinase (AKT), and signal transducer and activator of transcription 3 (STAT3) pathways, as well as processes related to extracellular matrix regulation and inflammatory responses. Importantly, *in vitro* and *in vivo* experiments demonstrated that RKIP overexpression sensitizes cells to anti‐EGFR treatments, whereas RKIP knockout diminished their sensitivity. Overall, our findings indicate that RKIP modulates LUAD progression and response to EGFR‐targeted therapies, although its clinical value as a biomarker requires further validation. These findings highlight RKIP's potential in overcoming therapeutic resistance and the need for further investigation into its regulatory mechanisms.

## Graphical abstract

RKIP, a metastasis suppressor protein, modulates key oncogenic pathways in lung adenocarcinoma. *In silico* analyses linked low RKIP expression to poor survival. Functional studies revealed RKIP overexpression reduces tumor aggressiveness and enhances sensitivity to EGFR‐targeted therapies, while its loss promotes resistance. RKIP expression has an impact on MAPK, AKT, and STAT3 signaling, supporting its potential as a biomarker and therapeutic modulator.

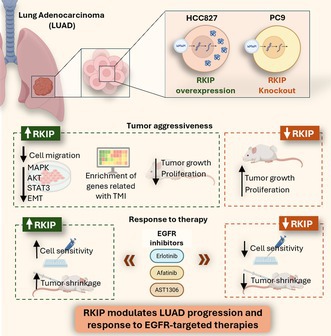

**Abbreviations**
AKT: RAC serine/threonine‐protein kinase
BACH1: BTB and CNC homology 1, basic leucine zipper transcription factor 1
CTR: control
DMEM: Dulbecco's modified Eagle's medium
DMSO: dimethyl sulfoxide
ECM: extracellular matrix
EGF: epidermal growth factor
EGFR: epidermal growth factor receptor
EMT: epithelial‐to‐mesenchymal transition
FBS: fetal bovine serum
GSK3β: glycogen synthase kinase 3 beta
IC_50_
: half maximal inhibitory concentration
IHC: immunohistochemistry
KO: knockout
KRAS: Kirsten rat sarcoma viral oncogene homolog
LUAD: lung adenocarcinoma
LUSC: lung squamous cell carcinoma
MAPK: mitogen‐activated protein kinase
MMP: matrix metalloproteinase
NF‐κB: nuclear factor kappa‐light‐chain‐enhancer of activated B cells
NSCLC: non‐small‐cell lung cancer
OE: overexpression
PBS: phosphate‐buffered saline
PEBP1: phosphatidylethanolamine‐binding protein 1 (gene encoding RKIP)
RKIP: Raf kinase inhibitory protein
RPMI: Roswell Park Memorial Institute (medium)
RT: room temperature
RTK: receptor tyrosine kinase
SD: standard deviation
STAT3: signal transducer and activator of transcription 3
TKI: tyrosine kinase inhibitor
TME: tumor microenvironment
WT: Wild‐type

## 1 Introduction

Lung cancer is the most commonly diagnosed cancer, with 2.48 million new cases every year, and is the leading cause of cancer‐related deaths, with more than 1.8 million new deaths per year among both genders [1, 2]. Effective public health measures to reduce smoking rates have significantly contributed to a reduction in lung cancer incidences, especially among men [3]. Although there have been some improvements in 5‐year survival rates, these have been modest, increasing only slightly over the past decade [4].

Histologically, lung cancer is classified into SCLC (small‐cell lung cancer) and NSCLC (non‐small‐cell lung cancer), accounting for 15% and 85% of cases, respectively. The latter is subdivided into several subtypes, with adenocarcinomas (LUAD) being the most common, accounting for 78% of cases [3]. Additionally, the genetic alterations within the tumors are determinants of the prognosis of the patients and the treatment strategies to be adopted. For the advanced stages, where surgery is no longer a viable option for treatment, molecular testing is performed, usually with a broad panel‐based approach to identify driver genetic alterations in genes such as *EGFR*, *KRAS*, *ALK*, *ROS1*, *RET*, *BRAF*, *MET*, and *NTRK* [5].

Alterations in these oncogenic drivers, typically mutually exclusive, lead to the constitutive activation of kinase signaling pathways [6, 7, 8]. Particularly, somatic activating mutations in *EGFR*, observed in 10–35% of NSCLC cases, were the first targets of tyrosine kinase inhibitors (TKis) approved for treating these patients [9]. The rise of first‐generation EGFR inhibitors, Gefitinib and Erlotinib, transformed lung cancer care. Yet, swift resistance prompted the development of second‐generation inhibitors, such as Afatinib, offering enhanced specificity [10]. Recently, third‐generation inhibitors, such as Osimertinib, targeting the T790M mutation, emerged to combat resistant *EGFR* mutations [11]. Despite the ongoing advancement of targeted therapies as an alternative to chemotherapy, resistance inevitably arises [12]. As EGFR signaling pathways play an important role in NSCLC [13], understanding their downstream signaling is crucial to better stratify patients, guide therapy choices, and provide insights into resistance mechanisms and putative overcoming strategies.

Raf kinase inhibitory protein (RKIP), also known as phosphatidylethanolamine‐binding protein 1 (PEBP1), is a multifunctional protein [14] that plays a pivotal role in various signaling pathways, including those directly or indirectly activated by receptor tyrosine kinases (RTK), such as the MAPK, NFκB, GPCR, and GSK3β cascades [14, 15]. RKIP's downregulation has been implicated in several cancers and was associated with metastasis occurrence and reduced overall survival [16]. In NSCLC, RKIP expression is reported to be low in 36–62% of the patients, which was associated with advanced disease stage, the presence of lymph node metastasis, and poor prognosis [17]. However, the studies on NSCLC remain limited, and the independent prognostic value of RKIP in NSCLC has not been established [17].

Given that RKIP is both a prognostic marker and a predictive indicator of patient response to conventional therapies [17], we hypothesize it could be a potential biomarker in NSCLC management. Thus, our study aimed to evaluate RKIP's impact on tumor aggressiveness and EGFR‐targeted therapy efficacy in NSCLC.

## 2 Materials and methods

### 2.1 *In silico* analysis of public lung cancer patients' datasets

All analyses of RKIP expression in NSCLC were conducted using the gene symbol “*PEBP1*.”

#### 2.1.1 Comparative analysis

A comparative analysis of *PEBP1* expression levels was done between normal and tumor tissue (normal vs tumor) using the Lung Cancer Explorer (LCE) database (https://lce.biohpc.swmed.edu/lungcancer/), a comprehensive repository of lung cancer data [18]. Our analysis focused on lung adenocarcinoma (LUAD) and lung squamous carcinoma (LUSC), the two primary subtypes of NSCLC. We utilized the TCGA_LUAD_2016 dataset (517 samples) for LUAD samples and the TCGA_LUSC_2016 dataset (501 samples) for LUSC samples.

#### 2.1.2 Survival analysis

Survival analysis was performed using the cBioPortal for Cancer Genomics platform (http://www.cbioportal.org) to assess the predictive value of *PEBP1* expression levels in patient survival. The analysis was performed separately for LUAD and LUSC using the Lung Adenocarcinoma dataset (TCGA, PanCancer Atlas) with 566 samples/patients and the Lung Squamous Cell Carcinoma dataset (TCGA, PanCancer Atlas) with 487 samples/patients, respectively. The samples were categorized into *RKIP* low (*PEBP1*: EXP < 0) and *RKIP* high (*PEBP1*: EXP > 0) groups based on mRNA expression levels measured by RNA Seq V2 RSEM, using a *z*‐score threshold of 0. Kaplan–Meier survival curves were generated in cBioPortal, and the Logrank test was applied with a significance threshold of < 0.05.

#### 2.1.3 Meta‐analysis

To validate our findings, we conducted meta‐analyses using the LCE platform, aggregating data from 56 studies comprising over 6700 lung cancer patients. First, a meta‐analysis of tumor *vs*. normal differential expression was performed, calculating the summary standardized mean difference (tumor‐normal) with Hedges' *G* as the effect size metric [18]. Meta‐analysis was conducted only for studies with at least 10 samples in each group and *PEBP1* gene data available from at least three qualifying studies. Hence, seven LUAD and seven LUSC studies were analyzed separately. Second, the survival meta‐analysis involved fitting Cox hazard models to assess the association between *PEBP1* gene expression and patient survival in each dataset [18]. The summary hazard ratio (HR) was derived from individual dataset HRs. For this analysis, 21 LUAD and 17 LUSC studies were considered separately.

### 2.2 Cell lines and cell culture

Four NSCLC cell lines were used in this work: H292 (RRID: CVCL_0455), kindly provided by Doctor Renato Silva (CPOM, Brazil), A549 (RRID: CVCL_0023) (University of Kent, UK), HCC827 (RRID: CVCL_2063) (University of Kent, UK), and PC9 (RRID: CVCL_B260) (ATCC, Manassas, VA, USA). All were grown in Dulbecco's Modified Eagle's Medium (DMEM, Biochrom GmbH, Berlin, Germany), except for PC9, which was maintained in Roswell Park Memorial Institute 1640 medium (RPMI‐1640 Medium; Biochrom GmbH). Both culture media were supplemented with 10% fetal bovine serum (FBS; Biochrom GmbH) and 1% penicillin/streptomycin (Gibco, Invitrogen, Carlsbad, CA, USA). All cell lines were cultured in a humidified atmosphere at 37 °C with 5% (v/v) CO_2_ and authenticated through short tandem repeat profiling. Additionally, regular testing was performed to ensure the absence of mycoplasma contamination in all cultures. All cell lines originated from the adenocarcinoma subtype, except for H292, which is histologically classified as being from mucoepidermoid pulmonary carcinoma. HCC827 and PC9 are *EGFR* mutants (exon 19 deletions), A549 is a *KRAS* mutant, and H292 is a wild‐type for *KRAS* and *EGFR*.

### 2.3 *In vitro* RKIP genetic modulation

For the generation of stable RKIP overexpression (OE), the pcDNA3 vector containing the full cDNA of RKIP, with the geneticin (G418) resistance gene, was used, which was kindly provided by Doctor Evan Keller (University of Michigan, USA). The HCC827 cell line was cultured in a six‐well plate at a density of 5 × 10^5^ cells per well and allowed to adhere overnight. FUGENE HD reagent (Roche, Basel, Switzerland) was used according to the manufacturer's protocol, with 2 μg of plasmid at a ratio of 6 : 2 (reagent : plasmid), in serum‐free Opti‐MEM media. After 24 h of incubation, the medium was replaced with a DMEM complete medium, and the cells were further selected with 500 μg·mL^−1^ of G418. Cells transfected with the empty vector were named HCC827 CTR, and RKIP overexpressing cells were named HCC827 RKIP OE.

For stable RKIP knockout (KO), a kit from Santa Cruz Biotechnology (Dallas, TX, USA) based on CRISPR/Cas9 technology was used. The PC9 cell line was cultured in a six‐well plate at a density of 5 × 10^5^ cells per well and allowed to adhere overnight. Cotransfection was done using the FUGENE HD reagent (Roche) according to the manufacturer's protocols, with 1 μg of RKIP CRISPR/CAS9 KO plasmid (sc‐401270‐KO‐2; Santa Cruz Biotechnology) and 1 μg of RKIP HDR plasmid (HDR Plasmid—sc‐401270‐HDR‐2; Santa Cruz Biotechnology) at a ratio of 6 : 2 (reagent : plasmids), in serum‐free Opti‐MEM media. After 48 h of incubation, the medium was replaced with a normal RPMI complete medium, and the cells were further selected with 0.5 μg·mL^−1^ of puromycin. Control cells were named PC9 CTR and knocked‐out ones as PC9 RKIP KO.

### 2.4 Immunofluorescence assay

A549, HCC827, H292, and PC9 cells were seeded on glass coverslips placed into 12‐well plates until ~ 60% confluence. Next, the cells were fixed and permeabilized in cooled methanol for 10 min. After blocking with 5% bovine serum albumin for 30 min, the cells were incubated overnight at room temperature (RT) with the primary antibody for RKIP (1 : 500, 07‐137; EMD Millipore, Burlington, MA, USA). After washing in phosphate‐buffered saline (PBS), the TRITC Alexa Fluor‐conjugated secondary antibody (Molecular Probes, Invitrogen, Waltham, MA, USA) was used at a dilution of 1 : 500 for 1 h at RT protected from light. Finally, after washing in PBS, cells were mounted in Vectashield Mounting Media with 4′,6‐diamino‐2‐phenylindone (Sigma‐Aldrich, St. Louis, MO, USA), and images were obtained with a fluorescence microscope (BX61, Olympus, Tokyo, Japan) at 200× magnification, using the cellsens software (Olympus, Tokyo, Japan).

### 2.5 Cell viability assay

To assess cellular viability over time, HCC827 and PC9 cells were seeded into 96‐well plates in triplicate at a density of 5 × 10^3^ per well and allowed to adhere overnight. The following day, fresh medium with 10% FBS was provided, and the cells were incubated for 24, 48, and 72 h. Day 1 was considered the 0‐h time point. The viable cells were quantified over time with the MTS assay‐Cell titer96 Aqueous cell proliferation assay (Promega, Madison, WI, USA). The results were calibrated to the starting value (time 0 h, considered 100% viability) and expressed as the mean ± SD. The assay was done in triplicate and at least three times.

### 2.6 Wound healing migration assay

HCC827 and PC9 cells were seeded in a six‐well plate and cultured to at least 95% confluence. Monolayer cells were washed with PBS, scraped with a plastic 1000 μL pipette tip, and incubated with fresh medium containing 10% FBS. The “wounded” areas were photographed by phase contrast microscopy at specific time points: for the HCC827 cells, it was 12, 24, 48, 72, and 84 h; for PC9 cells, it was 6, 12, 24, 32, and 36 h. The relative migration distance was calculated using the formula: percentage of wound closure (%) = 100 (*A* − *B*)/*A*, where *A* is the width of the cell wounds before incubation, and *B* is the width of the cell wounds after incubation. The results are expressed as the mean ± SD. The assay was done in triplicate at least three times.

### 2.7 Clonogenicity assay

HCC827 and PC9 cell lines were seeded in a six‐well plate at a density of 3000 cells per well and allowed to adhere overnight. The next day, fresh medium with 10% FBS was given, and cells were incubated for 10–15 days, with medium renewal every 3 days. The colonies were fixed with cold methanol for 10 min, stained with 0.05% crystal violet for 30 min, and manually counted. Results were expressed as the mean colonies ± SD. The assay was done in triplicate at least three times.

### 2.8 3D spheroids culture

PC9 and HCC827 tumor spheroids were obtained using the agarose coated‐overlay method [19]. In short, 96‐well plates were previously coated with 1.5% normal melting point agarose (NMP; Invitrogen—ThermoFisher Scientific, Carlsbad, CA, USA) in incomplete RPMI or DMEM medium, respectively. After solidification, PC9 cells (1.5 × 10^3^) and HCC827 (6 × 10^3^) cells were suspended in a complete medium and added to each well. The plates were transferred to the incubator and held immobile for 96 h for tumor spheroids formation (300–500 μm^3^), called the initiation phase.

The volume, morphology, and integrity of PC9 and HCC827 spheroids were analyzed based on the methods reported by Friedrich et al. [19] and Vinci et al. [20]. After the initiation phase, the spheroids were photographed using the Axio Cam MRc image capture system (Carl Zeiss, Göttingen, Germany) coupled to an inverted Axio LabA1 microscope (Carl Zeiss) using the 4× and 10× objectives. Integrity/morphology of the spheroids was assessed to detect irregular spheroids (without circular shape), cell disaggregation, or irregular cell agglomeration. The spheroid area was calculated using the imagej software (v1.8.0_345) (Washington, DC, USA).

These experiments were performed with six spheroids/replicates (*n* = 6) in three biological experiments.

### 2.9 Western blot analysis

The cells were seeded in a six‐well plate at a density of 1 × 10^6^ cells per well and allowed to adhere overnight. The next day, the cells were serum‐starved for 4 h. Before the end of the time point, cells were, when necessary, stimulated with 10 ng·mL^−1^ of Epidermal Growth Factor (EGF) for 15 min [21]. Protein quantification was performed using Bradford reagent (Sigma‐Aldrich, St. Louis, MO, USA). Aliquots of 50 μg of total protein from each sample were separated on a 10% polyacrylamide gel by sodium dodecyl sulfate/polyacrylamide gel electrophoresis (100 V) and transferred onto a nitrocellulose membrane (Amersham Biosciences, Amersham, UK) in 25 mm Tris‐base/glycine buffer using the Trans‐Blot Turbo Transfer System (25 V, 1 A for 30 min). The membranes were blocked with milk 5% Tris‐Buffered Saline/0.1% Tween (TBS‐Tween) for 1 h at RT and incubated overnight with the primary antibodies at 4 °C. The primary antibodies used are detailed in Table S1. After washing in TBS‐Tween, the membranes were incubated with the respective secondary antibodies coupled with horseradish peroxidase (1 : 2500; Cell Signaling Technology, Danvers, MA, USA). α‐Tubulin was used as a loading control. Blot detection was done by chemiluminescence (WesternBright Sirius; Advansta Inc., San Jose, CA, USA) using the Sapphire Biomolecular Imager (Azure Biosystems Inc., Dublin, CA, USA) and ChemiDoc™ XRS + System (Bio‐Rad, Hercules, CA, USA)).

### 2.10 mRNA NanoString™ analysis and functional enrichment analysis

Gene expression analysis on HCC827 wild‐type, CTR, and RKIP OE cells was performed using the NanoString nCounter PanCancer Pathways panel (730 gene transcripts) according to the manufacturer's instructions (NanoString Technologies, Seattle, WA, USA) [22]. The mentioned panel assesses 13 canonical pathways (Notch, Wnt, Hedgehog, Chromatin modification, Transcriptional Regulation, DNA Damage Control, TGF‐β, MAPK, STAT, PI3K, RAS, Cell Cycle and Apoptosis). For the hybridization process, 100 ng of RNA was used. Quality control parameters such as binding density, detection limit, positive controls, and housekeeping counts were measured using the nsolver™ Analysis Software v4.0 (NanoString Technologies) and ROSALIND®.

Assays presenting less than 30% of housekeeping genes above 50 counts were removed from the analysis. Raw data were normalized using housekeeping genes through the ROSALIND® platform, and absence was considered when counts were below 20, the background threshold level. The 25 genes differentially expressed between HCC827 WT and HCC827 CTR cells were excluded (data not shown). Quantile normalization and differential expression were performed within the nanostringnorm package (v1.0.0) in rstudio (v4.2.0). The normalized log_2_ mRNA expression values were used for subsequent data analysis. Genes with fold change (FC) ≤ −1.5 and > 1.5 and *P*‐value < 0.01 were considered significant. A heatmap with hierarchical clustering of differentially expressed genes was built with the complexheatmap package (v2.12.1).

A functional gene‐set enrichment analysis was performed with the list of genes differentially expressed between HCC827 CTR and HCC827 RKIP OE cells. Such was performed using the graphical gene‐set enrichment tool of ShinyGo V0.80 online platform (http://bioinformatics.sdstate.edu/go74/). The KEGG database was selected to identify which pathways were enriched upon RKIP overexpression, and an adjusted *P*‐value cutoff (FDR) of 0.05 was chosen as the significance threshold. Functional protein association network analysis was done using string v11.5 (https://string‐db.org) to assess the interaction between the enriched proteins. The protein–protein interaction (PPI) enrichment *P*‐value below 0.05 was considered to establish a significant enrichment of network interaction.

### 2.11 Pharmacological agents

Erlotinib, Afatinib, and AST1306 were obtained from Selleck‐Chemicals (Houston, TX, USA). All the agents were prepared as stock solutions in dimethyl sulfoxide (DMSO) and stored at −20 °C, as previously described [21]. In all experimental conditions, the drugs were diluted in 0.5% FBS culture medium. The vehicle control was also used in all experiments.

### 2.12 Cytotoxicity assay

To determine the concentration at which 50% of the cell viability is inhibited by drug treatment (half maximal inhibitory concentration—IC_50_), HCC827 and PC9 cells were seeded in 96‐well plates at a density of 5 × 10^3^ cells per well and allowed to adhere overnight in each respective complete medium. The cells were treated with increasing concentrations of the drugs or with DMSO alone, diluted in 0.5% FBS culture medium to a final concentration of 1% DMSO. After 72 h, cell viability was quantified using the Cell Titer96 Aqueous cell proliferation assay (Promega). The results were expressed as the mean percentage ± SD of viable cells relative to the DMSO alone (considered as 100% viability). The IC_50_ was calculated by nonlinear regression analysis using the graphpad prism software version 8 (Dotmatics, Boston, MA, USA).

### 2.13 *In vivo* mice studies

Immunodeficient NOD scid gamma (NSG) mice—NOD.Cg‐Prkdc scid Il2rg tm1Wjl /SzJ (Charles River Laboratories, Wilmington, MA, USA) were used for the *in vivo* studies. The mice were housed under standard laboratory conditions with a controlled 12‐h light/dark cycle, ambient temperature (21 ± 1 °C), and relative humidity of 50–60%. They had *ad libitum* access to irradiated food and autoclaved water. The health status of sentinel mice was monitored regularly to ensure they met the specified pathogen‐free criteria outlined by FELASA (Federation of European Laboratory Animal Science Associations). Throughout the experiments, the mice were observed periodically for signs of morbidity or mortality. Ethics approval for all mouse experiments was obtained from the institutional ethical committee (ORBEA, Portugal) and the Portuguese Directorate‐General for Food and Veterinary Affairs (DGAV, Portugal; reference DGAV 022524). All procedures were conducted in accordance with European Union Directive 2010/63/EU.

The subcutaneous xenograft mouse model assay was employed to assess the impact of RKIP on tumor growth and the potential inhibition of tumor growth by treatment with the TKI, Afatinib. Initially, either 1.5 × 10^6^ PC9 cells or 6 × 10^6^ HCC827 cells were injected into the right flank of 8–9‐week‐old female NSG mice. Tumors were allowed to develop, and their growth was measured biweekly using a caliper. Tumor volume was calculated using the formula: Volume = 3.14 (*D* ^2^ × *d*)/6, where *D* represents the major tumor axis, and *d* represents the minor tumor axis.

For the tumor growth assessment assays, the mice were euthanized, and the tumors were harvested after 57 and 21 days for HCC827 and PC9 cells, respectively. For assays involving TKI treatment, tumors were allowed to reach a medium size of approximately 200–300 mm^3^ before randomly assigning the mice to receive either vehicle (0.5% w/v methylcellulose/ 0.4% v/v tween80) or 10 or 15 mg·kg^−1^ of Afatinib, depending on the cell line used (HCC827 or PC9, respectively). Both the vehicle and Afatinib were prepared every 2 days and administered orally through a gavage once daily for 15 days. Tumor growth inhibition was evaluated by comparing the mean change in tumor volume between the control and treated groups from the start of treatment. After this treatment period, the experiment was concluded, and the tumors were harvested.

For both assays, the harvested tumors were either snap‐frozen in liquid nitrogen and stored at −80 °C for further analysis or fixed by immersion in 3.7% formaldehyde and subsequently embedded in paraffin for histological analysis.

### 2.14 RNA extraction, cDNA synthesis, and quantitative real‐time analysis

Total RNA was extracted directly from the tumor piece from the mice *in vivo* assays using TripleXtractor reagent (GRiSP Research Solutions, Porto, Portugal), according to the manufacturer's instructions. cDNA was synthesized from 1 μg of RNA using the Xpert cDNA Synthesis Kit (GRiSP Research Solutions), following the manufacturer's recommendations. *BACH1*, *RKIP*, and β‐actin (reference gene) expression levels were determined by qRT‐PCR using Xpert Fast SYBR 2x Master Mix (GRiSP Research Solutions). The reactions were performed using the 7500 Fast Real‐Time PCR software (Applied Biosystems, Waltham, MA, USA), under the following conditions: 95 °C for 30 s; 35 cycles of 95 °C for 5 s, 30 s at the respective annealing temperature. Primers sequences and annealing temperatures are described in Table S2. mRNA was normalized to β‐actin expression, according to $2^{-\Delta C_{t}}$ (*C*_t_: threshold cycle; presented as relative expression).

### 2.15 Immunohistochemistry analysis

Histological slides with 3‐μm‐thick tissue sections were subjected to immunohistochemical (IHC) analysis according to the streptavidin‐biotin peroxidase complex system (UltraVision Large Volume Detection System Anti‐Polyvalent, HRP; LabVision Corporation, Fremont, CA, USA), as previously described [21, 23]. Briefly, deparaffinized and rehydrated slides were submitted to heat‐induced antigen retrieval for 20 min at 98 °C with 10 mm citrate buffer (pH 6.0). For xenograft tissues, the primary antibodies used are detailed in Table S1. All the primary antibodies were incubated overnight at 4 °C. The secondary biotinylated goat anti‐polyvalent antibody was applied for 10 min, followed by incubation with the streptavidin‐peroxidase complex. The immune reaction was visualized by 3,3′‐diamonobenzidine (DAB) as a chromogen. All sections were counterstained with Gill‐2 hematoxylin. Stained slides were evaluated and photographed under a bright‐field microscope Olympus BX61 at 100× and 200× magnification using the cellsens software.

### 2.16 Statistical analysis

To determine statistical differences between groups in the *in vitro* and *in vivo* assays, single comparisons between the different conditions studied were made using Student's *t*‐test, and differences between groups were evaluated using the two‐way ANOVA test. Such was performed using graphpad prism 8 version. Statistical significance was defined as a *P*‐value less than 0.05.

## 3 Results

### 3.1 RKIP expression is significantly reduced in NSCLC and correlates with poor survival in lung adenocarcinoma: an *in silico* analysis

**Fig. 1:**
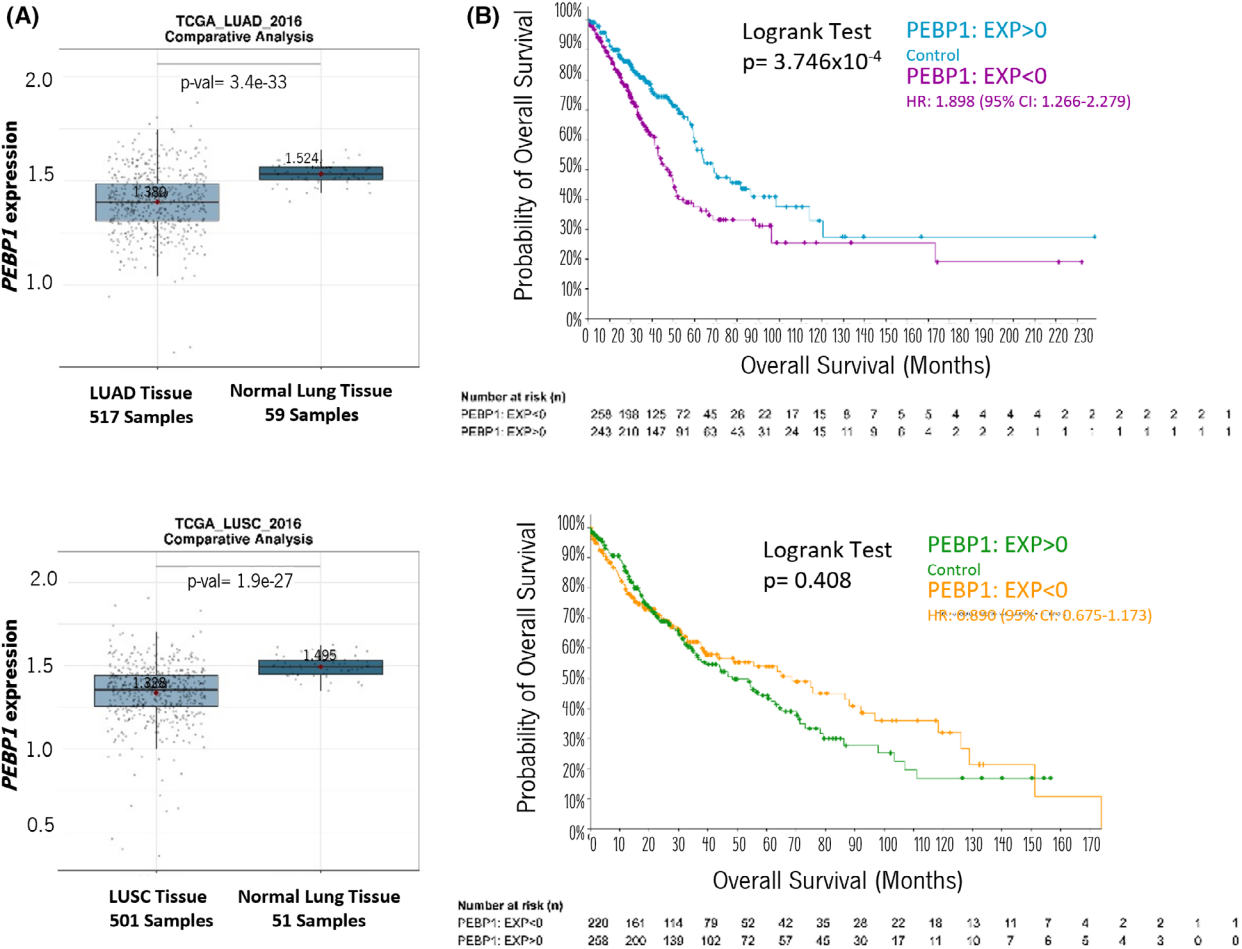
Correlation between *PEBP1* (RKIP) mRNA expression levels in normal and NSCLC tissues and its clinical relevance. (A) Comparative analysis of the *PEBP1* mRNA expression levels between LUAD tissues (517 samples) or LUSC tissues (501 samples) and normal lung tissues (59 or 51 samples), upper and lower panel respectively. Data were plotted on the LCE online platform from the TCGA_LUAD_2016 or TCGA_LUSC_2016 database. For the comparative analysis, the *t*‐test was used, and *P* < 0.05 was considered significant. Boxplots show the median and interquartile range (IQR); whiskers extend to data within 1.5 × IQR, and outliers are shown as individual points. (B) Kaplan–Meier distribution of LUAD (upper graph) and LUSC (lower graph) patient's overall survival (OS) in months, performed in cBioPortal. Patients were divided into two groups considering *PEBP1* expression levels: High (PEBP1: EXP > 0 (blue and green)) and Low (PEBP1: EXP < 0 (purple and yellow)). In LUAD's analysis, 258 samples and 243 were considered Low and High *PEBP1*, respectively. In LUSC's analysis, 220 samples and 258 were deemed Low and High *PEBP1*, respectively. For survival analysis, the Logrank test was performed, and *P* < 0.05 was considered significant. LUAD, lung adenocarcinoma; LUSC, lung squamous cell carcinoma; NSCLC, non‐small‐cell lung cancer; *PEBP1*, gene that encodes RKIP.

Using the Lung Cancer Explorer (LCE) platform, which aggregates data from multiple lung cancer patient databases, including TCGA, we independently investigated whether RKIP (*PEPB1* gene) expression varies among adenocarcinomas (LUAD) and squamous cell carcinomas (LUSC). A comparative analysis between normal lung tissue and LUAD or LUSC patients revealed a significant decrease in *PEPB1* expression in both tumor subtypes compared to normal surrounding lung tissue (Fig. 1A). Additionally, LCE provides a meta‐analysis tool that summarizes results across multiple studies while combining their statistical power. Hence, and as shown in the forest plots in Fig. S1, there is a consistent downregulation of *PEBP1* expression across LUAD studies (HR = −0.84; 95% CI, −1.15 to −0.54; *P* = 7.3e‐08) and LUSC studies (HR = −1.22, 95% CI; −1.49 to −0.94; *P* = 5.6e‐18).

Then, we explore whether *PEPB1* expression levels correlated with the overall survival (OS) and progression‐free survival (PFS) of TCGA patients. The samples were categorized into high (*PEBP1*: EXP > 0) and low (*PEBP1*: EXP < 0) expression groups, and we found in LUAD patients that higher levels of *PEBP1* were associated with increased OS (averaging 22.86 more months of survival) and PFS (an increase of 20.57 more months of survival) (Fig. 1B, Table 1, and Fig. S2). These results align with our meta‐analysis conducted through the LCE platform, which also highlighted a survival association with *PEPB1* expression in LUAD (HR = −0.76; 95% CI, 0.73–0.83; *P* < 0.01) but not in LUSC (HR = 0.99; 95% CI, 0.87–1.13; *P* = 0.89) across different studies (Fig. S3).

**Table 1:** Implication of *PEBP1* expression in the overall and progression‐free survival of LUAD and LUSC patients from TCGA database. CI, confidence interval; LUAD, lung adenocarcinoma; LUSC, lung squamous carcinoma; NA, not available.

Tumor subtype	Survival type	Groups	Total number of cases	Number of events	Median months (95% CI)	Logrank test *P*‐value
LUAD	Overall survival	Low *PEBP1* expression	258	107	36.44 (32.71–42.34)	**3.75E‐04**
		High *PEBP1* expression	243	74	59.11 (50.24–104.19)	
	Progression‐free	Low *PEBP1* expression	258	109	26.24 (22.75–37.61)	**0.0241**
		High *PEBP1* expression	243	94	47.11 (37.68–72.92)	
LUSC	Overall survival	Low *PEBP1* expression	220	88	65.58 (43.89–96.82)	0.408
		High *PEBP1* expression	258	115	48.33 (36.62–64.21)	
	Progression‐free	Low*PEBP1* expression	221	65	72.23 (57.76–NA)	0.616
		High *PEBP1* expression	258	74	75.78 (54.44–NA)	

*Note*: Bold values indicate statistically significant *P*‐values (*P* < 0.05).

This *in silico* analysis suggests a significant association between *RKIP* expression loss and worse survival, specifically in LUAD.

### 3.2 RKIP overexpression reduces cell migration and spheroid integrity via modulation of EGFR downstream pathways

**Fig. 2:**
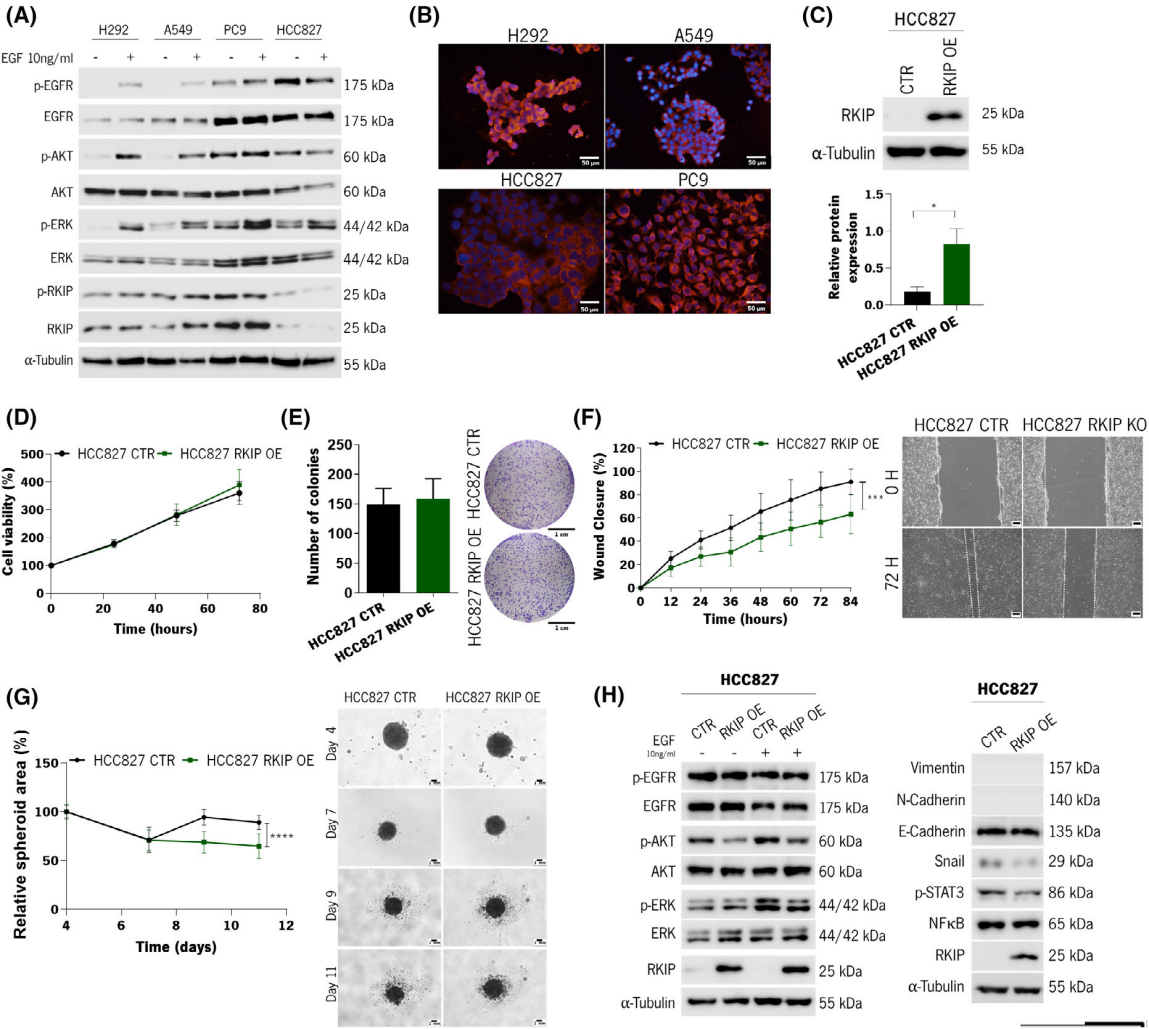
Evaluation of RKIP expression in NSCLC cell line, modulation of RKIP expression, and *in vitro* characterization of HCC827 RKIP overexpressing cell line. (A) Western blot analysis is used to assess the expression levels of the proteins EGFR, AKT, ERK, and RKIP and their respective phosphorylated forms in lung cancer cell lines, with and without EGF stimulation. This experiment was performed three times. (B) Immunofluorescence analysis of RKIP basal expression levels in lung cancer cell lines. These are representative pictures of two independent assays. Pictures were taken at 200× magnification. Scale bar: 50 μm. (C) Western blot analysis to assess the transfection efficiency to overexpress (OE) RKIP protein on the HCC827 cell line. On top is presented a representative image of three independent experiments, and on the bottom is shown the graphical representation of the quantification of the western blots. (D) Graphical representation of the cell's viability that was assessed at 24, 48, and 72 h using the MTS assay (*N* = 4). (E) Clonogenicity assay to assess both proliferation capacity and the capacity to form multicellular colonies after 14 days. Colonies were counted manually, and the representative pictures were taken using a stereomicroscope (Olympus SZ) at 1× magnification (*N* = 3). Scale bar: 1 cm. (F) Wound healing migration assay, where the capacity of the cells to migrate and close the wound was evaluated over time (12, 24, 36, 48, 60, 72, and 84 h) (*N* = 3). On the right are representative images taken at 100× magnification at 0 and 72 h. Scale bar: 200 μm. (G) Graphical representation of the cell's capacity to form spheroids over time (4, 7, 9, and 11 days). The spheroids area is represented in percentage, considering the spheroid size at day 4 at 100% (*N* = 3). Representative images are shown on the right and were acquired using the Axio LabA1 microscope at 100× magnification. Scale bar: 1 mm. (H) Western blot analysis to assess the expression levels of relevant proteins related to EMT, NFĸB, and EGFR signaling pathways in HCC827 RKIP OE and CTR cells. Regarding all western blots in this figure, cells were stimulated with EGF for 15 min at 10 ng·mL^−1^ when relevant and the experiments were performed three times. Also, α‐Tubulin was used as the loading control. In all graphics, error bars indicate the standard deviation (SD). Single comparisons between the different conditions studied were made using Student's *t*‐test, and differences between groups were evaluated using the two‐way ANOVA test. Values statistically different from the control group are represented with *P < 0.05, ***P < 0.001 and ****P < 0.0001. CTR, control; EGF, epidermal growth factor; NSCLC, non‐small‐cell lung cancer; RKIP OE, RKIP overexpression.

To explore the influence of RKIP in lung cancer cell aggressiveness, an initial characterization was done in four molecularly different NSCLC cell lines—H292, A459, PC9, and HCC827. The expression levels of RKIP and EGFR signaling players were assessed by western blot and immunofluorescence (Fig. 2A,B).

Western blot analysis showed that all cell lines express RKIP, although at distinct levels, with PC9 having the highest expression levels and HCC827 having the lowest levels (Fig. 2A). Similar trends were observed for the RKIP phosphorylated form (p‐RKIP) (Fig. 2A). Additionally, immunofluorescence analysis showed that RKIP is expressed mainly in the cytoplasm of the cells, although it might also be present in the nucleus of H292 cells (Fig. 2B).

Epidermal growth factor receptor was highly expressed and activated in *EGFR* mutant cell lines, PC9 and HCC827, as the remaining cell lines were less EGFR‐positive and activated only upon EGF stimulation (Fig. 2A). AKT and ERK were consistently significantly activated upon EGF stimulation in H292 and A549 cell lines, demonstrating that their survival is also dependent on the activation of receptors from the HER family. For PC9 and HCC827 cells, their constitutive activation of AKT and ERK pathways was verified, being the last one yet responsive to EGF stimulation (Fig. 2A).

Next, we selected HCC827 and PC9 cell lines, based on their relatively lower and higher RKIP expression levels, respectively, for *RKIP* gene modulation. RKIP was overexpressed (OE) on the HCC827 cell line and knocked out (KO) in the PC9 cell line, with successful transfection confirmed via western blot (Fig. 2C and Fig. S4A). The RKIP expression was amplified 4.8‐fold in HCC827 RKIP OE cells compared to control cells (Fig. 2C), while it was abolished entirely in PC9 RKIP KO cells (Fig. S4A).

HCC827 RKIP OE showed no significant differences in cell viability and clonogenicity capacity compared to the control cells (Fig. 2D,E). However, we found that cells overexpressing RKIP had a significantly lower migration capacity (Fig. 2F). As for PC9 cells, RKIP KO had no significant impact on the cells' proliferation, clonogenicity, and migration rates compared to the control cells (Fig. S4B–D).

The ability of the cells to form 3D spheroids was also evaluated. In the case of HCC827 cells, both clones formed tightly packed structures that tended to collapse around day 9, reducing the spheroid area over time (Fig. 2G). Although we did not observe an increase in spheroid size during the experiment, it is noteworthy that HCC827 RKIP OE spheroids showed a continuous decrease in area compared to their initial size, unlike HCC827 CTR spheroids (Fig. 2G). Thus, RKIP appears to be influencing spheroid integrity across the duration of the experiment. Conversely, PC9 cells formed fewer compact structures that exhibited significant growth (Fig. S4E). Still, no significant differences were observed between PC9 RKIP KO spheroid growth and the control ones (Fig. S4E).

Next, we sought to investigate the intracellular mechanisms in HCC827 cells, where the differences between the control and the RKIP‐modulated cells were more notorious. The western blot showed a decrease in the expression of the transcription factor Snail in HCC827 OE RKIP expression, a transcription factor considered central in the epithelial to mesenchymal transition (EMT) context (Fig. 2H). Other mesenchymal markers, N‐cadherin and Vimentin, were not expressed in the CTR cells or upon RKIP overexpression. As for the epithelial marker E‐cadherin, no significant differences were observed between clones. Furthermore, a decrease in the protein STAT3 (Tyr705) activation was also seen upon RKIP overexpression (Fig. 2H). No differences were seen regarding NFκB expression. Regarding the activation levels of EGFR‐related proteins, with and without stimulation of the receptor by EGF, no significant differences were observed in the activation levels of EGFR, which is not surprising as the receptor is mutated and, thus, constitutively activated. However, there was a significant decrease in the activation of ERK and AKT, both at basal and stimulated conditions, in the cells RKIP OE when compared with RKIP CTR cells (Fig. 2H). Given the described RKIP‐BACH1 feedback loop in breast cancer, we also assessed BACH1 protein and mRNA expression levels in our models. However, RKIP modulation did not result in significant alterations in BACH1 expression (Fig. S5).

Overall, the *in vitro* functional characterization unveiled significant differences upon RKIP overexpression and suggested that RKIP may modulate LUAD aggressiveness by inhibiting EGFR downstream pathways, particularly MAPK, AKT, and STAT3.

### 3.3 RKIP overexpression alters key genes involved in cancer progression and immune regulation

**Fig. 3:**
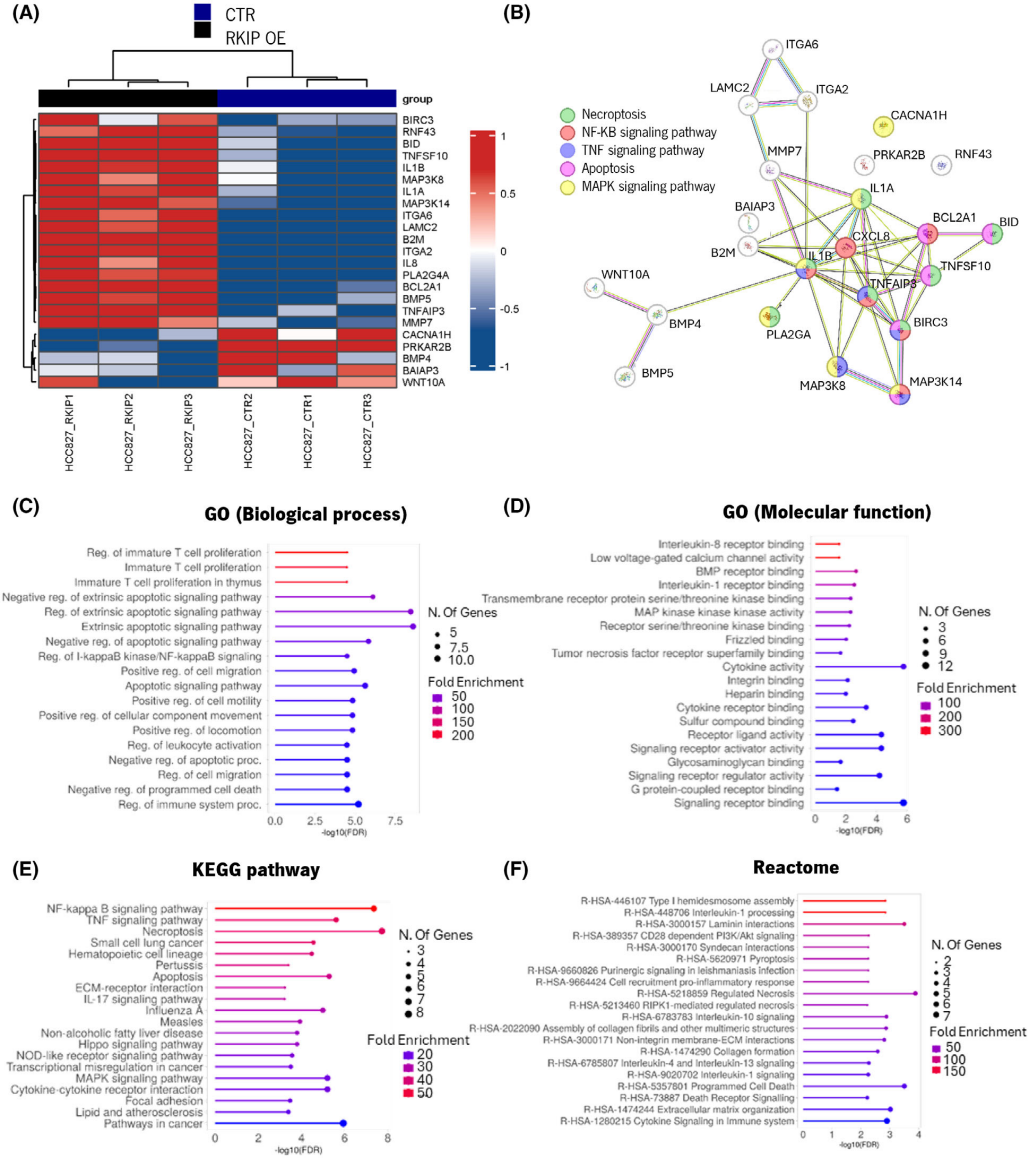
Molecular characterization of HCC827 cells after RKIP overexpression through NanoString analysis. (A) Heatmap of genes altered in HCC827 CTR (dark blue) and HCC827 RKIP OE cells (black). Red represents the overexpressed genes and in blue the downregulated genes. *P* adjusted < 0.01; fold‐change (FC) ≥ 1.5. (B) Functional protein association network associated with RKIP overexpression done in STRING, using the list of differentially expressed genes from NanoString analysis. The network formed had a significant interaction among proteins (PP1 enrichment *P*‐value = 1.11e^−16^). In this representation, each circle represents a protein (node), and each connection represents a direct or indirect connection (edge). Node color indicates the pathway that these proteins are related to green—necroptosis, red—NFKB pathway, purple—tumor necrosis factor (TNF) pathway, pink—apoptosis, and yellow—MAPK pathway. Line color indicated the type of interaction evidence: purple—experimental evidence, light blue—curate database, black—co‐expression, pink—experimentally determined, yellow—text mining, dark blue—gene co‐occurrence. (C–F) The differential expressed genes between HCC827 RKIP OE and CTR cells were also explored on ShinyGO platform through a functional enrichment analysis. Gene Ontology (GO) (C, D), KEGG (E), and Reactome (F) databases were used, and the top 20 pathways enriched considering our group of genes were compiled and represented. Enriched pathways were sorted considering the −log_10_ (FDR), the size of the circles is proportional to the number of genes, and the color of the bars corresponds to the fold enrichment.

We further conducted a differential transcriptomic analysis to identify alterations in gene expression induced by RKIP overexpression. Using the NanoString PanCancer Pathways Panel, we evaluated the gene expression profiles between HCC827 cells (CTR *vs*. OE). We observed 23 differentially expressed genes between CTR and RKIP OE cells (Fig. 3A). Of these, 18 genes were overexpressed (*BIRC3*, *RNF43*, *BID*, *BMP5*, *TNFSF10*, *BCL2A1*, *IL1B*, *B2M*, *MAP3K8*, *ITGA*, *IL1A*, *IL8*, *MAP3K14*, *PLA2G4A*, *ITGA6*, *TNFAIP3*, *LAMC2*, *MMP7*) and five genes were downregulated (*CACNA1H*, *BMP4*, *PRKAR2B*, *BAIAP3*, *WNT10A*) when RKIP expression was induced (Fig. 3A).

A comprehensive analysis of the 23 differentially expressed genes was conducted to explore their functional roles using the ShinyGO v0.80 platform (Fig. 3C,D). Additionally, pathway enrichment analysis was performed to identify common regulatory pathways affected by RKIP overexpression in HCC827 cells (Fig. 3E,F). We found enrichment in pathways associated with immune response activation, regulation of T cell proliferation, apoptotic signaling activation, regulation of cell migration, extracellular matrix organization, as well as receptor activity and signaling transduction pathways such as NF‐κB, MAPK, and PI3K/AKT.

Furthermore, the STRING functional protein association network analysis revealed several interactions (PPI enrichment *P*‐value: 1.11e^−16^), with 20 out of 23 proteins having at least two connections (Fig. 3B). Notably, proteins encoded by the genes *IL1B*, *TNFAIP3*, *IL8*, *BIRC3*, *MAP3K14*, *CXCL8*, *MAP3K8*, *TNFSF10*, and *BCL2A1* formed central nodes, indicating their involvement in necroptosis, NF‐κB, tumor necrosis factor (TNF), apoptosis, and MAPK signaling pathways.

These results provide valuable insights into the molecular mechanisms influenced by RKIP overexpression in lung cancer models.

### 3.4 RKIP expression modulates *in vivo* tumor growth

**Fig. 4:**
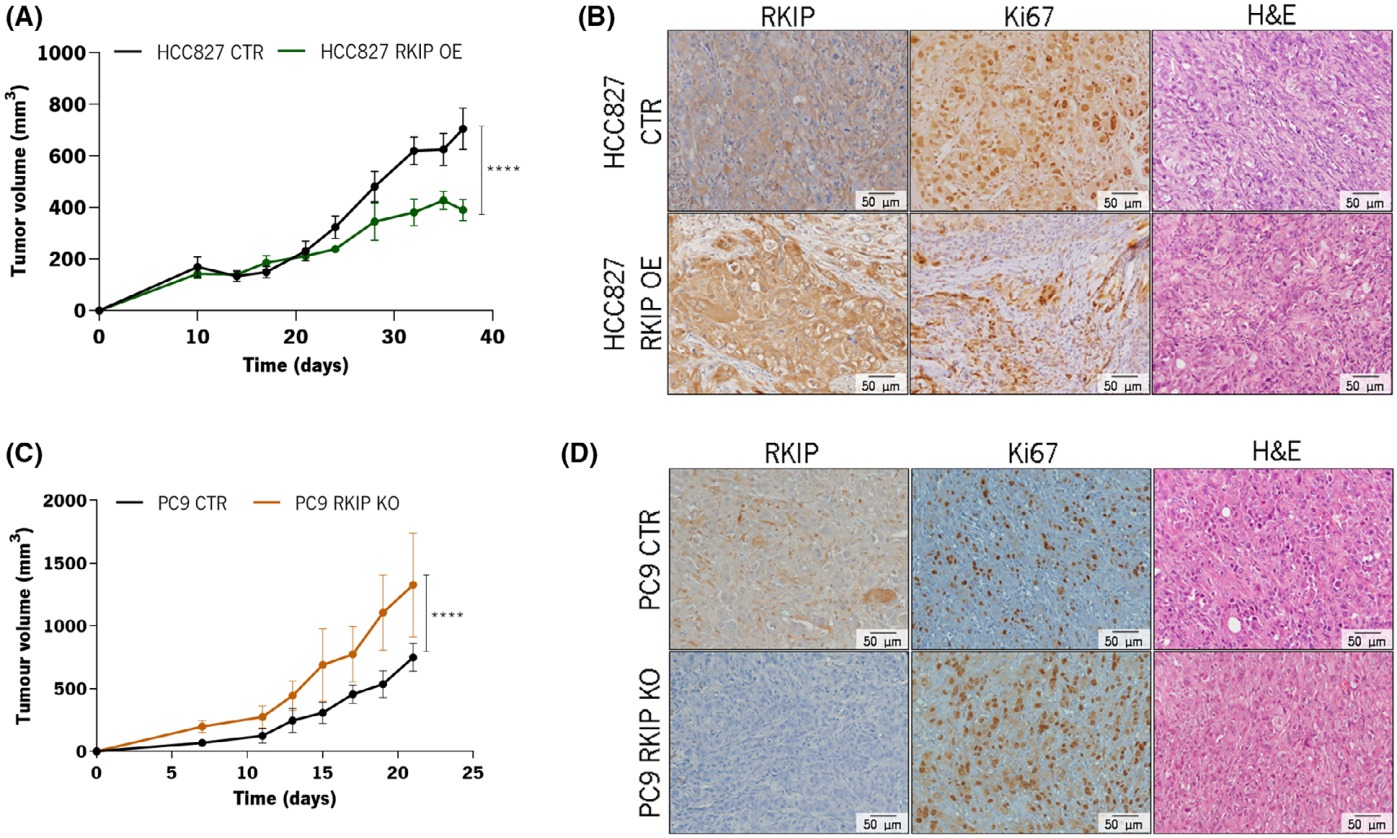
*In vivo* assessment of the influence of RKIP modulation in tumor growth. (A) Effect of RKIP overexpression in tumor growth rate upon injection of HCC827 RKIP OE and CTR cells in immunocompromised mice. Tumors were manually measured with a caliper over time for 37 days (*N* = 8). (B) Representative immunohistochemistry pictures of the expression of RKIP, the proliferation marker Ki67, and H&E staining of RKIP CTR and RKIP OE tumors (*N* = 3). (C) Effect of RKIP knockout in tumor growth rate upon injection of PC9 RKIP KO and CTR cells in immunocompromised mice. Tumors were manually measured with a caliper overtime for 21 days (*N* = 8). (D) Representative immunohistochemistry pictures of the expression of RKIP, the proliferation marker Ki67 and H&E staining of RKIP CTR and RKIP KO tumors (*N* = 3). In all graphics, error bars indicate the standard deviation (SD) and the differences between groups were evaluated using the two‐way ANOVA test. Values statistically different from the control group are represented with ****P < 0.0001. CTR, control; H&E, hematoxylin and eosin staining; RKIP KO, RKIP knockout; RKIP OE, RKIP overexpression.

To further explore our results, *in vivo* experiments were performed to evaluate RKIP's influence on LUAD's tumorigenesis. Overexpressing RKIP significantly suppressed subcutaneous tumor growth in mice (Fig. 4A), accompanied by lower expression of the proliferation marker Ki67 (Fig. 4B). Interestingly, in the PC9 RKIP KO model, despite minimal differences observed *in vitro*, inhibiting RKIP expression conferred a growth advantage to tumors compared with control tumors *in vivo* (Fig. 4C). This effect was further supported by increased Ki67 marker staining observed in PC9 KO tumor tissues (Fig. 4D).

These findings also underscore the significance of studying RKIP's role in an *in vivo* model. Our data emphasize the importance of considering the tumor microenvironment, as RKIP knockout exhibited minimal impact *in vitro* but had a significant effect when assessed *in vivo*.

### 3.5 RKIP expression modulates the response to EGFR inhibitors

RKIP has demonstrated influence in the response to chemotherapeutic drugs across various tumor types. Thus, given the prominence of targeted therapies, particularly anti‐EGFR agents, in LUAD treatment and considering RKIP's observed modulation of pathways downstream of this receptor, we investigated whether RKIP expression could affect cellular response to EGFR‐targeted therapies.

**Fig. 5:**
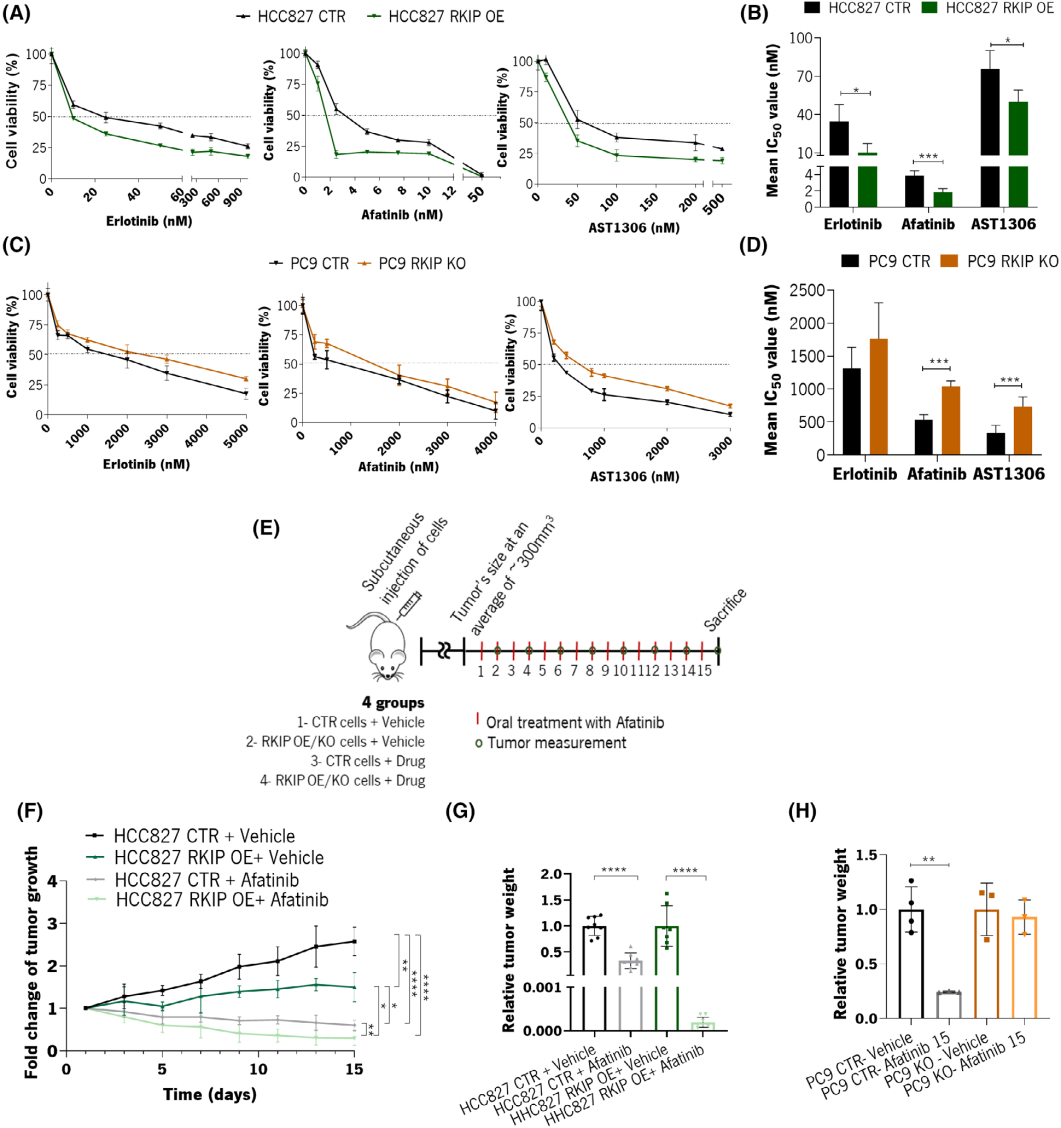
Effect of RKIP modulation in cells response to EGFR‐targeted therapies, *in vitro* and *in vivo*. (A) Representative graphics of the cytotoxicity assays used for the anti‐EGFR drugs, Erlotinib, Afatinib, and AST1306, in HCC827 RKIP OE and CTR cells. Such was assessed for 72 h by MTS assay. The graphs represent the mean ± SD relative to DMSO alone (100%) viability. These are representative of four independent assays performed in triplicate. (B) Comparative IC_50_ values analysis for HCC827 CTR and RKIP OE cells. IC_50_ values are the mean of four independent assays performed in triplicate. (C) Representative graphics of the cytotoxicity assays used for the anti‐EGFR drugs, Erlotinib, Afatinib and AST1306, in PC9 RKIP KO and CTR cells. Such was assessed for 72 h by MTS assay. The graphs are represented as the mean ± SD, relative to DMSO alone (100%) viability. These are representative of four independent assays performed in triplicate. (D) Comparative analysis of IC_50_ values for PC9 KO and CTR cells. IC_50_ values are represented as the mean of four independent assays performed in triplicate. (E) Scheme of the *in vivo* experiment with Afatinib treatment upon subcutaneous injection of HCC837 RKIP OE or PC9 RKIP KO and respective control cells. (F) Relative tumor growth rate upon daily treatment with 10 mg·kg^−1^ of Afatinib by oral gavage (*N* = 8). Tumor growth of all animals was normalized, taking into consideration the tumor size on the first day of treatment. (G) Representation and comparative analysis of the final tumor weight. The tumor weight of the nontreated animals was considered 1 to normalize the samples (*N* = 8). (H) Representation and comparative analysis of the final tumor weight upon daily treatment with 15 mg·kg^−1^ of Afatinib by oral gavage. In this, the tumor weight of the non‐treated animals was considered as 1 to normalize the samples (*N* = 3). In all graphics, error bars indicate the standard deviation (SD). Single comparisons between the different conditions studied were made using Student's *t*‐test, and differences between groups were evaluated using the two‐way ANOVA test. Values statistically different from the control group are represented with *P < 0.05, **P < 0.01, ***P < 0.001 and ****P < 0.0001. CTR, control; RKIP KO, RKIP knockout; RKIP OE, RKIP overexpression.

To do so, we treated the genetically modified *RKIP* cell models with Erlotinib and Afatinib, 1st‐ and 2nd‐generation anti‐EGFR agents, respectively. It was also tested AST1306, an analog of Afatinib [24]. *In vitro* cytotoxicity assays revealed that HCC827 RKIP OE cells exhibited increased sensitivity to these drugs compared to control cells, as evidenced by lower cell viability over time and reduced IC_50_ concentrations (Fig. 5A,B and Table 2). Conversely, PC9 RKIP KO cells demonstrated decreased sensitivity to all three anti‐EGFR drugs tested (Fig. 5C,D and Table 2).

**Table 2:** Mean IC_50_ values for Erlotinib, Afatinib, and AST1306 in HCC827 RKIP OE, PC9 RKIP KO, and respective control cells. The IC_50_ values are presented in nm ± SD.

Mean IC_50_	HCC827 (nm)		PC9 (nm)	
	RKIP CTR	RKIP OE	RKIP CTR	RKIP KO
Erlotinib	34.63 ± 11.44	10.29 ± 6.34	1317 ± 274	1763 ± 471
Afatinib	3.87 ± 0.53	1.83 ± 0.39	527 ± 68	1036 ± 76
AS1306	75.69 ± 12.47	50.10 ± 7.90	333 ± 109	734 ± 132

Furthermore, HCC827 RKIP OE and PC9 RKIP KO cells and their respective control cells were injected subcutaneously into mice, followed by daily treatment with Afatinib (Fig. 5E). Treatment efficacy with 10 mg·kg^−1^ of Afatinib significantly reduced tumor growth, a tendency that is evident in the downward trend of the gray and light green lines presented in Fig. 5F. On the other hand, tumors derived from HCC827 RKIP OE cells exhibited a higher shrinkage rate over time (Fig. 5F), reducing relative tumor weights than tumors from HCC827 CTR cells (Fig. 5G). Conversely, tumors derived from PC9 RKIP KO cells showed less responsiveness to treatment with 15 mg·kg^−1^ of Afatinib, as evidenced by the absence of tumor shrinkage over time compared to treated PC9 CTR tumors (Fig. 5H).

Therefore, these *in vitro* and *in vivo* findings reveal that RKIP levels shape LUAD cells' response to EGFR‐targeting drugs.

## 4 Discussion

Despite significant advancements in lung cancer therapeutic options, such as tyrosine kinase inhibitors, resistance to treatment continues to pose a major challenge in lung cancer management [4, 5, 12]. Therefore, identifying biomarkers capable of predicting patients' prognosis and response to therapy, enabling patient stratification and optimized treatment selection, remains critically important in oncology. In this study, we investigate the influence of RKIP expression on NSCLC aggressiveness and its influence on response to therapy. RKIP has been studied in various malignancies and is recognized as a metastasis suppressor [16]. Yet, the extent of its loss and its implications for both lung cancer aggressiveness and patient outcomes remain unclear [17].

We conducted an *in silico* analysis using TCGA, which revealed a significant reduction in *RKIP* (*PEBP1*) expression in tumor tissues—LUAD and LUSC—compared to normal tissues. Notably, the clinical relevance of RKIP loss appears subtype‐specific: reduced *RKIP* expression correlates with poorer overall and progression‐free survival in LUAD, but not in LUSC. In fact, a few studies explored the effect of RKIP on LUSC, and the results were unclear [25, 26].

Therefore, we focused on LUAD to interrogate how RKIP influences aggressiveness. *RKIP* was genetically modulated in two LUAD cell lines, and the *in vitro* results revealed that overexpressing RKIP significantly impaired migration and spheroids' integrity, consistent with its proposed tumor‐suppressive function [27]. However, this effect was not as evident in PC9 cells, where no major differences were observed *in vitro*. These discrepancies are not unexpected, as different cell lines and *in vitro* conditions may mask RKIP's true influence. Similar discrepancies between *in vitro* and *in vivo* findings have been reported in other tumor models, often due to the influence of the tumor microenvironment (TME), which is absent in traditional cell culture conditions [28, 29]. Notwithstanding, *in vivo* experiments provided clearer evidence of RKIP's impact on tumor growth, as discussed below. Given these findings, we prioritized HCC827 cells for the initial biological characterization of RKIP in LUAD.

The overexpression of RKIP on HCC827 cells was associated with a downregulation of oncogenic pathways such as MAPK, AKT, and STAT3. This inhibitory role of RKIP has been reported in NSCLC and other cancers [15, 17] but not in these lung cancer cells, which are *EGFR* mutants. Additionally, we observed an inhibitory effect of RKIP in Snail expression, a transcription factor central to the EMT process. Wang et al. [30] reported similar findings, with RKIP overexpression affecting migration and invasion rates via EMT modulation. However, our study broadens this knowledge by analyzing two EGFR‐mutant cell lines, unlike most RKIP studies in lung cancer, which focus on *KRAS*‐mutant A549 cells [31, 32], thus expanding the understanding of RKIP's relevance in EGFR‐mutant settings, which account for 10–30% of NSCLC cases [9].

When exploring the influence of RKIP overexpression in gene expression, our transcriptomic analysis of HCC827 RKIP OE cells revealed alterations in novel genes modulated by RKIP in lung cancer. Further gene enrichment analysis performed from this list revealed alterations in pathways related to immune response regulation, cell proliferation, and extracellular matrix organization—aligning with RKIP's known functions, particularly seen in breast cancer models [33, 34, 35, 36, 37].

Among the genes altered by RKIP modulation, and besides the ones present in our transcriptomic analysis, we also explored *BACH1* expression, given its reported feedback loop with RKIP in breast cancer [38, 39, 40]. In our LUAD models, BACH1 expression remained unchanged at both the mRNA and protein levels upon RKIP overexpression, suggesting that this interaction may not be as relevant in EGFR‐mutant LUAD, reinforcing RKIP's tumor‐specific roles. Furthermore, our gene enrichment analysis did not highlight EMT as a predominant pathway influenced by RKIP, further supporting the notion that the RKIP‐BACH1 axis may not play a central role in EGFR‐mutant LUAD. While further research is warranted to elucidate these mechanisms, our findings suggest that RKIP's tumor‐suppressive effects in EGFR‐mutant LUAD may be mediated through alternative pathways beyond BACH1‐EMT regulation.

*In vivo* studies reinforced RKIP's role in tumor modulation, revealing a modulatory effect in PC9 cells not observed *in vitro*. HCC827 RKIP OE cells suppressed tumor growth, while PC9 RKIP KO cells significantly accelerated it in mice. The discrepancy between *in vitro* and *in vivo* results suggests that RKIP's interaction with the TME may be contributing to these observations. Although the immunodeficient mouse model used in this work lacks most immune cells, other TME components could have contributed to the observed effects. RKIP has been implicated in regulating immune cell infiltration, secretion of prometastatic factors, and expression of matrix metalloproteinases (MMPs) involved in extracellular matrix remodeling [28, 35, 41]. Additionally, RKIP has been shown to influence cytokine signaling, which may further impact the tumor–stroma crosstalk [41, 42]. While its role in LUAD remains to be fully characterized, our findings, alongside transcriptomic data, suggest that RKIP may influence LUAD progression through interactions beyond tumor‐intrinsic mechanisms, warranting further investigation.

Next, we explored whether RKIP expression could modulate cellular sensitivity to EGFR inhibitors and if its loss could influence these treatments' efficacy. Our results showed that RKIP expression levels influence the sensitivity of the cells to anti‐EGFR drugs, namely, Erlotinib (1st generation), Afatinib, and AST1306 (2nd generation). Overexpressing RKIP increased drug sensitivity by 1.5‐ to 3‐fold compared to the control cells and, on the other hand, upon depletion of RKIP expression, cells sensitivity reduced by two‐fold compared to control cells. These results were further validated *in vivo*, specifically with treatment with Afatinib.

RKIP's role in therapy response has been explored in various cancers, where it has been implicated in modulating sensitivity to conventional treatments [16, 27]. In NSCLC, chemotherapeutic agents such as adriamycin [43], 9‐nitrocamptothecin (9NC) [43], gemcitabine [44], and cisplatin [45] have been reported to upregulate RKIP as part of their mechanism of action, promoting apoptosis. Additionally, RKIP expression has been positively associated with radiosensitivity in NSCLC through modulation of the SHH signaling pathway and Gli1 expression [32].

Nonetheless, RKIP's influence on targeted therapy response remains poorly understood. In NSCLC, Giovannetti et al. [46] reported that Erlotinib treatment upregulated RKIP, likely via NF‐κB inhibition, while sorafenib (a multi‐TKI) slightly reduced RKIP expression. The combination of these agents demonstrated strong synergy in lung cancer cell lines, possibly due to RKIP‐mediated suppression of ERK and AKT activation, leading to increased apoptosis [46]. Similarly, our group previously observed that Erlotinib‐mediated EGFR inhibition increased RKIP expression in cervical cancer cells [47]. Still, in this study, *in silico* data revealed a negative correlation between EGFR mRNA/protein levels and RKIP mRNA expression in both LUSC and LUAD, suggesting a regulatory interaction between RKIP and EGFR [47]. This inverse relationship may contribute to RKIP's role in modulating sensitivity to EGFR inhibitors observed in this study.

While further research is needed to clarify the mechanisms underlying RKIP's impact on targeted therapy response, this study paves the way for exploring RKIP as a potential modulator of LUAD sensitivity to anti‐EGFR treatments and a biomarker of therapy response.

## 5 Conclusions

In conclusion, we report for the first time that RKIP significantly influences LUAD cell responses to EGFR inhibitors. Our findings also reinforce RKIP's role in regulating cell migration through the modulation of key pathways, including MAPK, AKT, STAT3, and EMT. Additionally, this study highlights RKIP's potential impact on the TME, although further research is needed to clarify the mechanisms by which RKIP regulates tumor aggressiveness through the TME and influence therapeutic responses. These could be important in overcoming resistance in LUAD. Finally, validating RKIP's clinical value as a biomarker for prognosis and EGFR therapy response in patient datasets remains a priority for future studies.

## Conflict of interest

The authors declare no conflict of interest.

## Author contributions

AR‐C, RMR, and OM contributed to conceptualization; AR‐C, JP, PF, INFG, ACL, and RJS‐O contributed to methodology; AR‐C, RFM, and JP contributed to in silico analysis; AR‐C, JP, RFM, PF, DC‐C, AM, RMR, and OM contributed to investigation; AR‐C contributed to writing—original draft preparation; AR‐C, JP, RFM, PF, DC‐C, AM, INFG, ACL, RJS‐O, RMR, and OM contributed to writing—review and editing; RMR and OM contributed to supervision, project administration, and funding acquisition; all authors have read and agreed to the published version of the manuscript.

## Peer review

The peer review history for this article is available at https://www.webofscience.com/api/gateway/wos/peer‐review/10.1002/1878‐0261.70096.

## Supporting information


**Fig. S1.** Forest plot results of the meta‐analysis of *PEBP1* expression between tumor vs normal tissue in LUAD and LUSC studies.
**Fig. S2.** Correlation between *RKIP* mRNA expression levels and clinical relevance in NSCLC tissues.
**Fig. S3.** Forest plot results of the *PEBP1* survival meta‐analysis in LUAD and LUSC studies.
**Fig. S4.** Evaluation of RKIP expression in NSCLC cell lines, modulation of RKIP expression and *in vitro* characterization of PC9 RKIP knocked out cell line.
**Fig. S5.** Assessment of BACH1 protein and mRNA expression levels upon RKIP modulation in HCC827 overexpression and PC9 knockout models.
**Table S1.** Details of the primary antibodies used for western blot and immunohistochemistry.
**Table S2.** Sequence of primers used for qRT‐PCR studies.

## Data Availability

Data presented in the study are available upon request to the corresponding author.
