# Supplementary material for: RKIP overexpression reduces lung adenocarcinoma aggressiveness and sensitizes cells to EGFR‐targeted therapies
Source: Mol Oncol. 2025 Jul 28;19(11):3205–22. doi: 10.1002/1878-0261.70096 (PMC12591310; doi:10.1002/1878-0261.70096)
Supplement: Supplementary file 1 — Fig. S1. Forest plot results of the meta‐analysis of PEBP1 expression between tumor vs normal tissue in LUAD and LUSC studies. Fig. S2. Correlation between RKIP mRNA expression levels and clinical relevance in NSCLC tissues. Fig. S3. Forest plot results of the PEBP1 survival meta‐analysis in LUAD and LUSC studies. Fig. S4. Evaluation of RKIP expression in NSCLC cell lines, modulation of RKIP expression and in vitro characterization of PC9 RKIP knocked out cell line. Fig. S5. Assessment of BACH1 protein and mRNA expression levels upon RKIP modulation in HCC827 overexpression and PC9 knockout models. Table S1. Details of the primary antibodies used for western blot and immunohistochemistry. Table S2. Sequence of primers used for qRT‐PCR studies. [file MOL2-19-3205-s001.pdf]

SUPPORTING INFORMATION

Figures

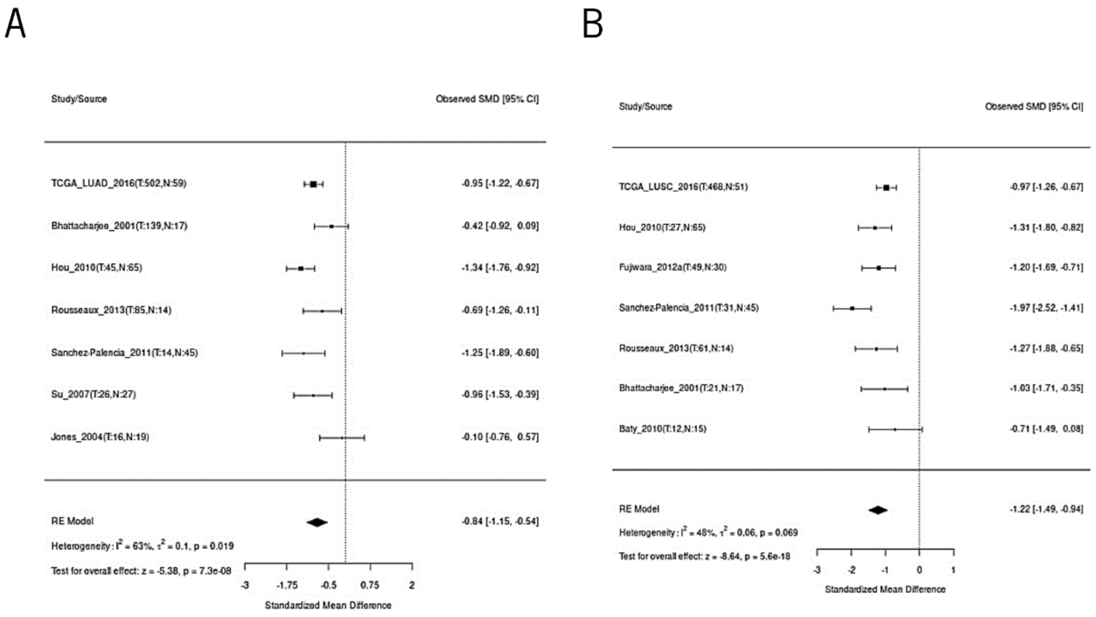

Fig. S1- Forest Plot results of the Meta-analysis of *PEBP1* expression between tumor vs normal tissue in LUAD (A) and LUSC (B) studies. Six studies were used for this analysis and in each forest plots, the name of each study is followed by the number of tumor and normal samples. The horizontal scale used is logarithmic. SMD: standardized mean difference; HR: hazard ratio; CI: confidence interval; LUAD: Lung Adenocarcinoma; LUSC: Lung squamous cell carcinoma.

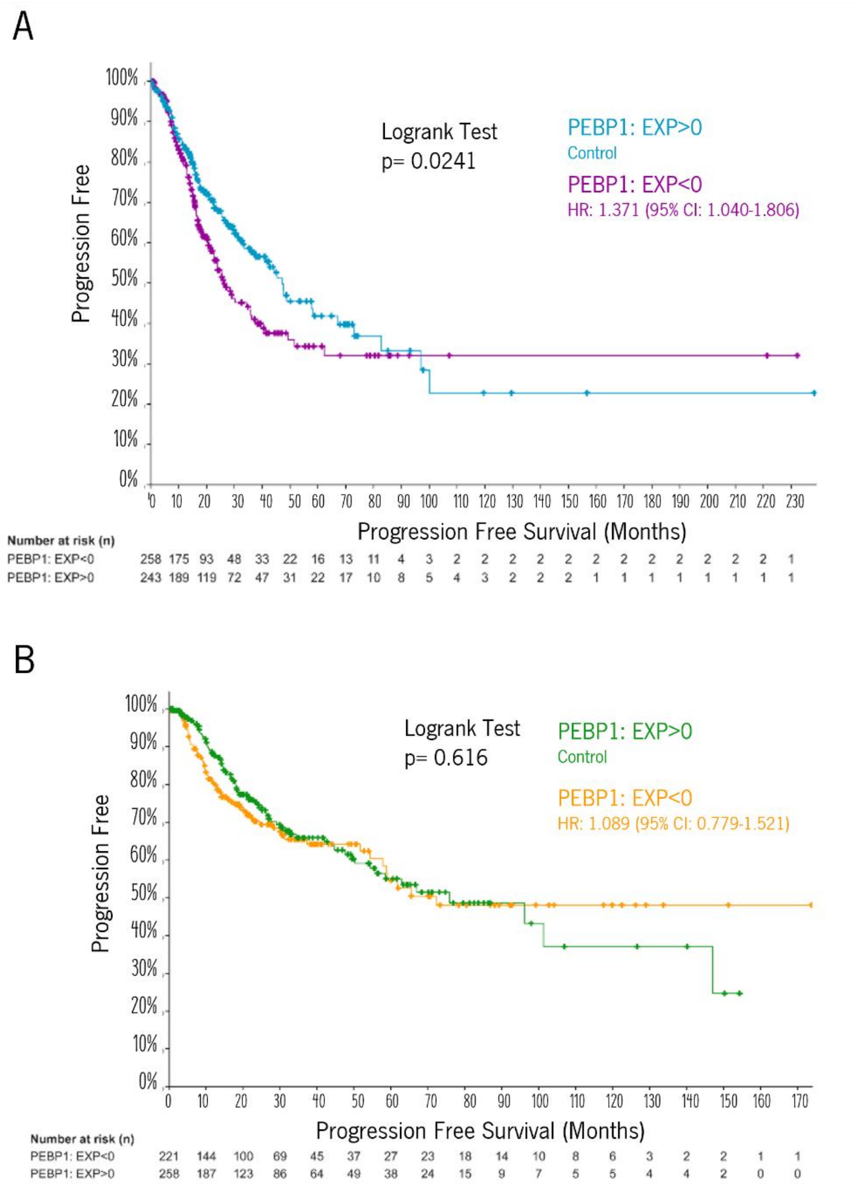

Fig. S2 - Correlation between *PEBP1* mRNA expression levels and clinical relevance in NSCLC tissues. Kaplan-Meier plots showing progression-free survival (PFS) in months for LUAD **(A)** and LUSC **(B)** patients, analyzed using cBioPortal. Patients were divided into two groups based on *PEBP1* expression levels: High (PEBP1: EXP > 0; blue and green) and Low (PEBP1: EXP < 0; purple and yellow). For LUAD, 258 samples were classified as Low *PEBP1* and 243 as High *PEBP1*. For LUSC, 221 samples were classified as Low *PEBP1* and 258 as High *PEBP1*. Survival

analysis was performed using the Log-rank test, with $p < 0.05$ considered significant. PEBP1: gene encoding RKIP; LUAD: Lung Adenocarcinoma; LUSC: Lung Squamous Cell Carcinoma.

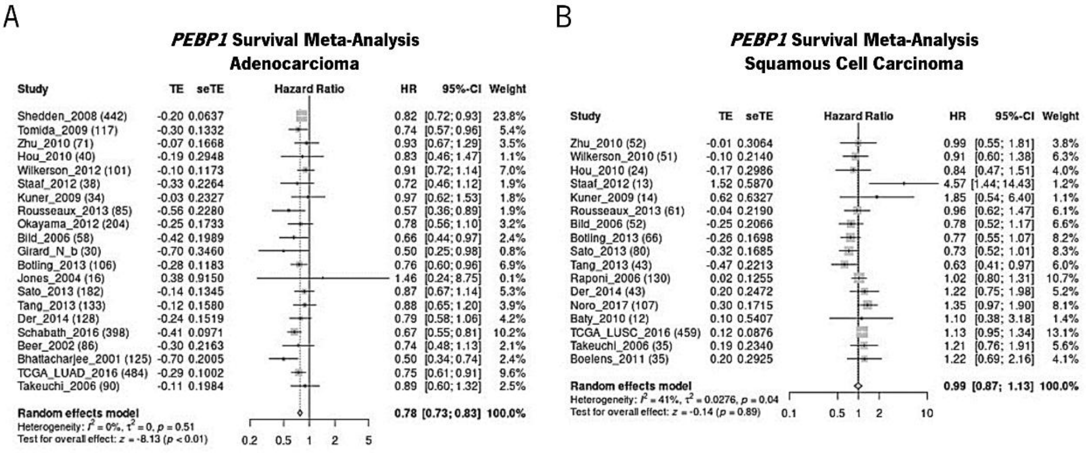

Fig. S3- Forest Plot results of the *PEBP1* survival meta-analysis in LUAD (A) and LUSC (B) studies. 21 LUAD studeis and 17 LUSC studies were evaluated and in each forest plots, the name of each study is followed by the number of tumor samples. The horizontal scale used is logarithmic. SMD: standardized mean difference; TE: estimated effect; sTE: standard error of estimated effect; HR: hazard ratio; CI: confidence interval; LUAD: Lung Adenocarcinoma; LUSC: Lung squamous cell carcinoma.

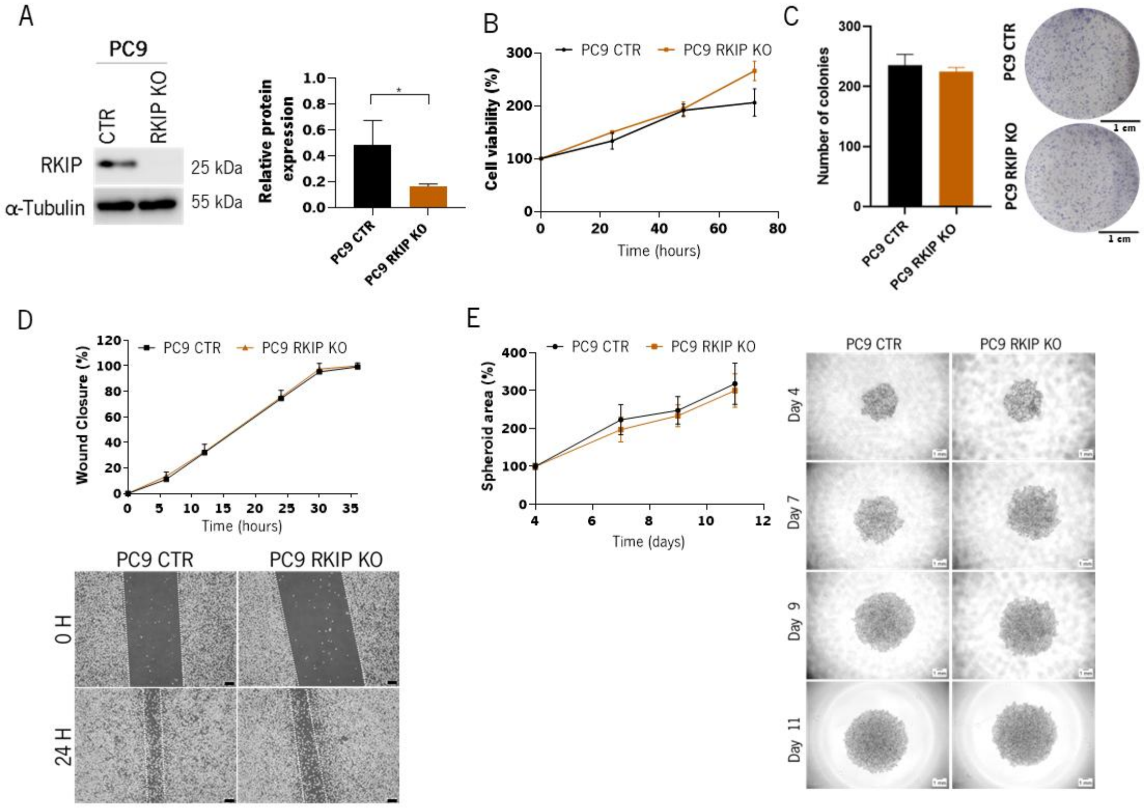

Fig. S4 - Evaluation of RKIP expression in NSCLC cell lines, modulation of RKIP expression and *in vitro* characterization of PC9 RKIP KO cell line. A) Western Blot analysis to assess the transfection efficiency to knockout (KO) RKIP protein on PC9 cell line. On the left side is presented a representative image of three independent experiments, and on the right side is shown the graphical representation of the quantification of the western blot. **B)** Graphical representation of the cell's viability assessed at 24, 48 and 72 hours by MTS assay (N=3). **C)** Clonogenicity assay to assess both proliferation capacity and the capacity of cells to form multicellular colonies after 14 days. Colonies were counted manually, and the representative pictures were taken using a stereomicroscope (Olympus SZ) at 1x magnification (N=3). Scale bar: 1 cm. **D)** Wound healing migration assay, where the capacity of the cells to migrate and close the wound was evaluated overtime (6, 12, 24, 30 and 36 hours) (N=5). On the bottom are representative images at 0 and 24 hours. Scale bar: 200 μm. **G)** Graphical representation of the cells capacity to form spheroids overtime (4, 7, 9 and 11 days) (N=3). The spheroids area is

represented in percentage, considering the spheroid size at day 4 the 100%. Representative images are shown on the right and were acquired using the Axio LabA1 microscope at 40x magnification. Scale bar: 1 mm. Single comparisons between the different conditions studied were made using Student's t-test, and differences between groups were evaluated using the two-way ANOVA test. Values statistically different from the control group are represented with * ($p<0.05$). RKIP KO: RKIP Knockout; CTR: Control.

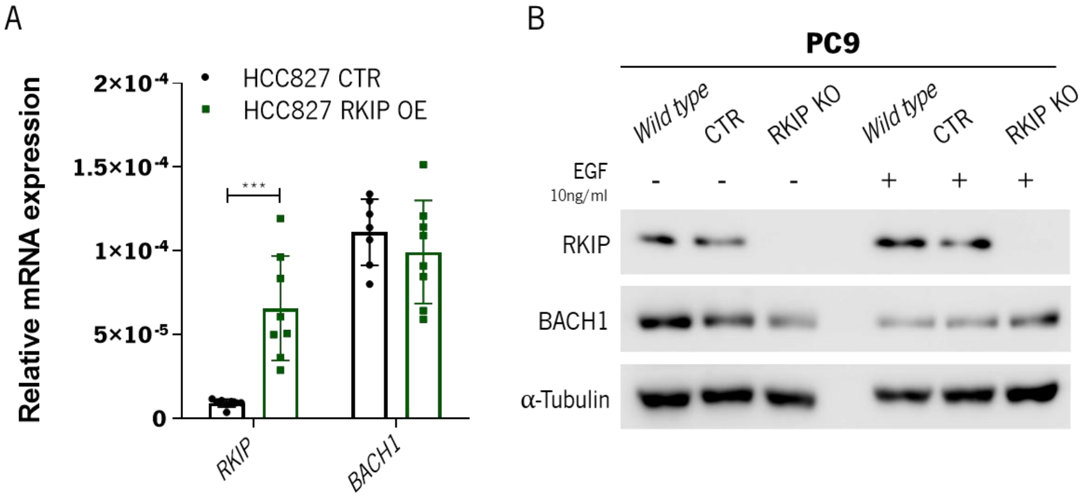

Fig. S5- Assessment of BACH1 protein and mRNA expression levels upon RKIP modulation in HCC827 overexpression and PC9 KO models. A) qRT-PCR analysis to assess the mRNA expression levels of the genes *RKIP* and *BACH1* of mice tumors derived from HCC827 CTR and HCC827 RKIP OE cell lines. The results were calibrated to β-actin, used as the reference gene. All the experiments were performed at least 3 times with three replicates. Values statistically different from the control group are represented with *** $p<0.001$. **B)** Western Blot analysis to assess the expression levels of RKIP and BACH1 in PC9 wild type, CTR and RKIP KO cells. Cells were stimulated with EGF for 15 minutes at 10ng/ml. α-Tubulin was used as the loading control.

Tables

Table S1- Details of the primary antibodies used for Western Blot and immunohistochemistry.

Antibody	Product reference	Technique	Incubation
AKT	4691, Cell Signaling	WB	1:1000, ON, 4°C
BACH1	SC271211, Santa Cruz Biotecnhology	WB	1:1000, ON, 4°C
E-caderin	3195, Cell Signaling	WB	1:1000, ON, 4°C
EGFR	4267, Cell Signaling	WB	1:1000, ON, 4°C
ERK1/2	4695, Cell Signaling	WB	1:1000, ON, 4°C
N-Cadherin	13116, Cell Signaling	WB	1:1000, ON, 4°C
NFκB	8242, Cell Signaling	WB	1:1000, ON, 4°C
p-AKT (Ser473)	9018, Cell Signaling	WB	1:1000, ON, 4°C
p-EGFR (Tyr1068)	3777, Cell Signaling	WB	1:1000, ON, 4°C
p-ERK1/2 (Trh202/tyr204)	4695, Cell Signaling	WB	1:1000, ON, 4°C
p-GSK3β(Ser9)	9323, Cell Signaling	WB	1:1000, ON, 4°C
p-STAT3 (Tyr705)	9145, Cell Signaling	WB	1:1000, ON, 4°C
RKIP	13006, Cell Signaling	WB	1:1000, ON, 4°C
Snail	3879, Cell Signaling	WB	1:500, ON, 4°C
Vimentin	5741, Cell Signaling	WB	1:1000, ON, 4°C
p-RKIP (Ser153)	SC135779, Santa Cruz Biotechnology	WB	1:1000, ON, 4°C
α-tubilin	SC23948, Santa Cruz Biotechnology	WB	1:1000, ON, 4°C
RKIP	sc-376925, Santa Cruz Biotechnology	IHC	1:600, ON, 4°C
ki67	PA5-16785, Invitrogen	IHC	1:200, ON, 4°C

(WB: Western Blot; IHC: Immunohistochemistry; ON: Overnight).

Table S2- Sequence of primers used for qRT-PCR studies.

Transcript target	Sense Primer	Antisense Primer	Annealing temperature (°C)
BACH1	CACCGAAGGAGACAGTGAATCC	GCTGTTCTGGAGTAAGCTTGTGC	58
β-*actin*	GGACTTCGAGCAAGAGATGG	AGCACTGTGTTGGCGTACAG	58
RKIP	GACATCAGCAGTGGCACAGT	GTCACACTTTAGCGGCCTGT	58
